# Prolyl Isomerase Pin1 Directly Regulates Calcium/Calmodulin-Dependent Protein Kinase II Activity in Mouse Brains

**DOI:** 10.3389/fphar.2018.01351

**Published:** 2018-11-23

**Authors:** Taiki Shimizu, Kenta Kanai, Yui Sugawara, Chiyoko Uchida, Takafumi Uchida

**Affiliations:** ^1^Molecular Enzymology, Department of Molecular Cell Science, Graduate School of Agricultural Science, Tohoku University, Sendai, Japan; ^2^Department of Human Development and Culture, Fukushima University, Fukushima, Japan

**Keywords:** prolyl isomerase, Pin1, tau, CaMKII, tauopathy

## Abstract

Calcium/calmodulin-dependent protein kinase II (CaMKII) is abundant in the brain and functions as a mediator of calcium signaling. We found that the relative activity of CaMKII was significantly lower in the WT mouse brains than in the Pin1^-/-^ mouse brains. Pin1 binds to phosphorylated CaMKII and weakens its activity. For this reason, the phosphorylation level of tau in the presence of Pin1 is lower than that in the absence of Pin1, and microtubule polymerization is not downregulated by CaMKII when Pin1 is present. These results suggest a novel mechanism of action of Pin1 to prevent neurodegeneration.

## Introduction

The peptidyl prolyl *cis/trans* isomerase Pin1 binds to phosphorylated Ser/Thr-Pro and isomerizes the peptide bond. Pin1^-/-^ mice (Fujimori et al., 1999) had Alzheimer’s disease-like phenotypes (Liou et al., 2003; Xu et al., 2017). However, the relationship between Pin1 and Alzheimer’s disease remains unclear (Akiyama et al., 2005; Pastorino et al., 2006). We have previously performed GST-Pin1 pull down analysis using mouse brains to determine the proteins that interact with Pin1 in the brain, and we identified several proteins including histone, synapsin, and CaMKII (Tatara et al., 2010). We speculate that Pin1 controls various neuronal functions associated with the CaMKII signaling pathway.

Calcium/calmodulin-dependent protein kinase II is abundant in the brain. It functions as a mediator of calcium signaling, and plays an important role in synaptic plasticity, which affects learning and memory (Lisman et al., 2002). It has been reported that the overexpression of CaMKII inhibits neuronal differentiation in PC12 cells (Massé and Kelly, 1997), and that constitutive CaMKII activation impairs the development of cholinergic synapses in giant fibers (Kadas et al., 2012). Dysregulation of CaMKII has been observed in neurological diseases including ADHD (Yabuki et al., 2014), Parkinson’s disease (Zaichick et al., 2017), and Alzheimer’s disease (Ghosh and Giese, 2015). These reports suggested that adequately regulating CaMKII in the brain is important for preventing neuronal dysfunction.

One of the substrates of CaMKII is tau, a microtubule-associated protein that presents mainly on axons in neuronal cells and promotes microtubule polymerization (Drechsel et al., 1992). The function and stability of tau are regulated by several kinds of modifications, such as acetylation, glycation, ubiquitination, and phosphorylation (Mietelska-Porowska et al., 2014). Phosphorylation is the most important modification that regulates tau function. In general, phosphorylation decreases tau activity (Johnson and Stoothoff, 2004). Proper regulation of tau phosphorylation is very important for microtubule control; the accumulation of hyperphosphorylated tau aggregates and neuronal loss are observed in the brains of tauopathy patients (Šimić et al., 2016).

In the present paper, we showed that Pin1 binds to CaMKII directly and weakens its relative activity, which reduces the level of phosphorylated tau. When there is a high level of tau phosphorylation, microtubule polymerization is not regulated properly. These results suggest a novel mechanism of action of Pin1 to prevent neurodegeneration.

## Experimental Procedures

### Animals

Pin1^-/-^ mice were generated and bred according to the methods described in our previous report (Fujimori et al., 1999). Our study was approved by the Tohoku University Animal Use and Care Committee, and all investigations were conducted according to the principles of the Declaration of Helsinki. Genotypes of the mice bred by mating Pin1^+/-^ mice were examined using the polymerase chain reaction (PCR). Primers: WILD1.2A (5-AAG GGA TTA GAA GCA AGA TTC G-3), 2L (5-AGC ACC CGA TCC TGT TCT GCA A-3′), and Start2 (5-CAG AGG CCA CTT GTG TA-3′) (Fujimori et al., 1999).

### Cell Culture

Neuro2a (N2a) cells were cultured in Dulbecco’s modified Eagle’s medium (DMEM; Nacalai Tesque Inc.) supplemented with 10% fetal bovine serum (FBS) and 1% penicillin/streptomycin. The cells were grown in 5% CO_2_ at 37°C, and transfected with the following plasmids as previously demonstrated (Shimizu et al., 2016): *Pin1 WT, Pin1 mutant W34A* (WW mutant: Pin1 without WW domain function), *R68/69A* (PPIase mutant: Pin1 without substrate affinity), and *flag-CaMKIIα*. Transfection was performed using Lipofectamine 2000 (Qiagen).

### Western Blotting

The cells were lysed in sample buffer (62.5 mM Tris–HCl, 5% sucrose, 2% sodium dodecyl sulfate (SDS), 5% β-mercaptoethanol, pH 6.8). The samples were analyzed by sodium dodecyl sulfate-polyacrylamide gel electrophoresis and western blotting using the following antibodies: anti-CaMKII antibody (1:1000) (H-300; Santa Cruz), anti-Pin1 antibody (1:2000) (Cell Signaling Technology, Danvers, MA, United States), Phospho-Tau Ser416 antibody (1:1000) (Cell Signaling Technology), Phospho-Tau Ser262 antibody (1:2000) (Thermo Fisher Scientific), and Tau5 antibody (1:2000) (BD Biosciences) for total tau. LAS-3000 (Fujifilm) was used for detection. MultiGauge software (Fujifilm) was used to measure the bands semi-quantitatively.

### CaMKII Kinase Assay

Mouse brains were lysed in extraction buffer (100 mM piperazine-N,N′-bis(2-ethanesulfonic acid) (PIPES), pH 6.9; 10 mM ethylenediamine tetraacetic acid (EDTA); 10 mM ethylene glycol-bis(β-aminoethyl ether)-N,N,N′,N′-tetraacetic acid (EGTA); 1 mM phenylmethylsulfonyl fluoride (PMSF); 2 mM Na_3_VO_4_; 2 mM NaF; 2 mM dithiothreitol (DTT); protease inhibitor cocktail (Nacalai Tesque Inc.). The N2a cells were transiently transfected with the *flag-CaMKIIα* and *Pin1* (*WT, W34A, R68/69A*) plasmids. After 24–48 h, the cells were lysed in the extraction buffer. The cell lysates were analyzed using a CaMKII Assay Kit (Promega). The substrate peptide of this kit contains no Ser/Thr-Pro site to which Pin1 can bind. ATP-γ-32P (3000 Ci/mmol, 10 mCi/mL; PerkinElmer). The expression level of the CaMKII proteins was measured semi-quantitatively from the intensities of western blot bands using MultiGauge software (Fujifilm).

The relative activity (kinase activity/protein level) was determined from these experimental results. Recombinant CaMKIIα derived from baculovirus (Carna Biosciences Inc.) was incubated with recombinant Pin1 for 1 h. The activity was determined using a CaMKII Assay Kit (Promega) with ATP-γ-^32^P (3000 Ci/mmol, 10 mCi/mL; PerkinElmer) or a western blotting investigation of tau phosphorylation at Ser262.

### Microtubule Polymerization Assay

10 μM of tau was phosphorylated by 10 nM CaMKIIα with or without 40 nM Pin1 for 3 h or phosphorylated by 50 nM CaMKIIα with or without 100 nM Pin1 for 14.5 h. These samples were directly added to the microtubule polymerization assay mixtures (Uchida et al., 2009; Gotoh et al., 2013). The optical density of the polymerized microtubules solution was measured at 350 nm (*A*_350_).

### Statistical Analysis

Results were reported as mean ± SD. Statistical significance was determined by Student’s *t* test or Dunnett’s test (multiple test). We evaluated that the value of *p* < 0.05 was statistically significant.

## Results

### Comparison of CaMKII Activity, Protein Levels, and Specific Activity Between Wild-Type (WT) and Pin1^-/-^ Mouse Brains

**FIGURE 1:**
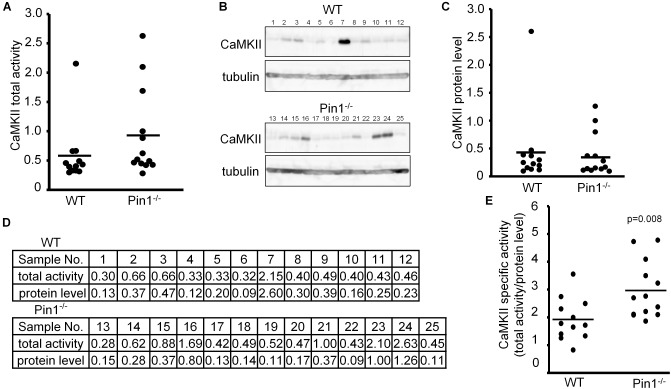
Activity and expression levels of CaMKII in wild-type (WT) and Pin1^-/-^ mouse brains. **(A)** CaMKII total activities in WT and Pin1^-/-^ mouse brain lysates (*n* = 12 and 13 distinct mouse brains, respectively) were analyzed using a CaMKII Assay Kit with ATP-γ-32P, and the relative values were plotted. **(B)** Western blot analysis of CaMKII. Tubulin was used as a loading control. **(C)** Relative expression levels of CaMKII in the brain lysates of WT and Pin1^-/-^ mice. The western bands were measured semi-quantitatively from the intensities of western blot bands using MutiGauge software. *p* = 0.8 using the Student’s *t*-test (not significant difference). **(D)** Relative values of CaMKII total activities and protein levels in WT and Pin1^-/-^ mouse brain lysates. **(E)** Each CaMKII-specific activity in the WT and Pin1^-/-^ mouse brain lysates was estimated by dividing the relative value of the total activity by the relative value of the protein level. *p* = 0.008 using the Student’s *t*-test.

We compared CaMKII kinase activity and CaMKII expression levels among brain lysates of WT and Pin1^-/-^ mice (*n* = 12 and 13 mice, respectively). Because of numerical variations between mice, there was not significant difference between them but the total CaMKII kinase activity in the Pin1^-/-^ mouse brains tended to be higher than that in the WT mouse brains (Figure 1A). The CaMKII expression levels did not differ significantly between the two (Figures 1B,C). The values obtained from these analyses are summarized in Figure 1D. The specific CaMKII activities (total kinase activity/protein level) were calculated and compared between the WT and Pin1^-/-^ mouse brains. The specific activity of CaMKII was significantly lower (*p* = 0.008) in the WT brains than in the Pin1^-/-^ brains (Figure 1E).

### Comparison of Phosphorylated Tau Between WT and Pin1^-/-^ Mouse Brains

**FIGURE 2:**
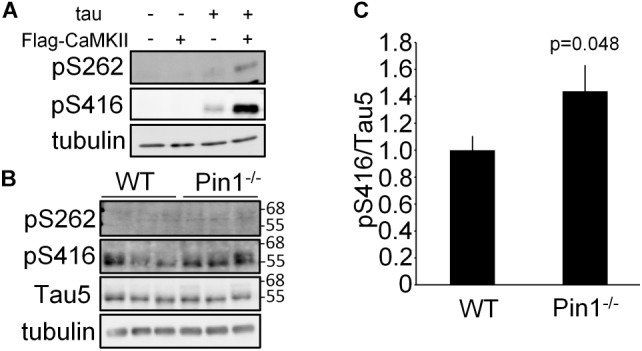
Phosphorylation levels of tau at Ser262 and Ser416 in wild-type (WT) and Pin1^-/-^ mouse brains. **(A)** Western blot analysis of phosphorylation of tau by CaMKII in N2a cells. *tau* and *flag-CaMKIIα* plasmids were transfected into N2a cells. The lysates were used to determine phosphorylation of tau by CaMKII using anti-pSer262 antibody and anti-pSer416 antibody. **(B)** Western blot analysis a of pSer262-tau and pSer416-tau in brain lysates of WT and Pin1^-/-^ mice (*n* = 3 mice, respectively). Total tau was detected using Tau5 antibody. Tau5 membrane was used for detection of pSer416 after inactivation of HRP by H_2_O_2_. Tubulin was used as a loading control. **(C)** Relative pSer416/Tau5 values. ^∗^*p* < 0.05 using the Student’s *t*-test.

It has previously been reported that Ser262 of tau is phosphorylated by CaMKII and it has been reported to be associated with Alzheimer’s disease (Seubert et al., 1995; Singh et al., 1996). In addition to Ser262, it has also been reported Ser416 is phosphorylated by CaMKII (Yamamoto et al., 2005). First, it was confirmed that the levels of phosphorylated Ser262 tau and phosphorylated Ser416 tau were increased in the N2a cells transfected with *CaMKIIα* cDNA (Figure 2A). Next, the phosphorylated Ser262 and phosphorylated Ser416 tau levels in the brain lysates from WT and Pin1^-/-^ mice (*n* = 3 mice, respectively) were compared. Phosphorylated Ser262 was not detected in either of brains, but the phosphorylated Ser416 was detected (Figure 2B). The value of phosphorylated Ser416 tau/total tau was significantly higher in the Pin1^-/-^ mouse brains than in the WT mouse brains (Figures 2B,C). These results indicate that Pin1 regulates CaMKII activity in mouse brains.

### Pin1 Suppressed CaMKII Activity

**FIGURE 3:**
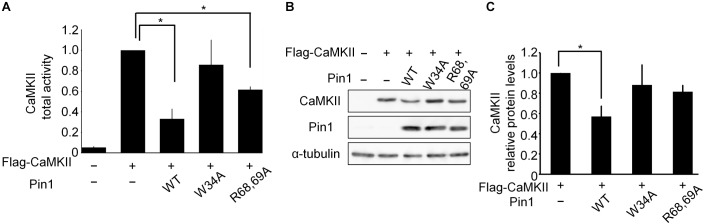
CaMKII activity and expression levels in Neuro2a cells. **(A)** CaMKII total activity in N2a cells. A *flag-CaMKIIα* plasmid was transfected with *mock* or *pin1* plasmids into N2a cells. The lysates were used to determine CaMKII total activity. The data represent the means ± SDs of three independent experiments. ^∗^*p* < 0.05 using Dunnett’s test. **(B)** Protein levels of CaMKII in N2a cells were analyzed by western blotting after transfection of the *flag-CaMKIIα* plasmid with *mock* or *pin1* plasmids. Tubulin was used as a loading control. **(C)** Relative values of CaMKII protein levels in panel **(B)**. The data represent means ± SDs of three independent experiments. ^∗^*p* < 0.05 using Dunnett’s test.

We examined the effect of Pin1 on CaMKII total activity in N2a cells. N2a cells transfected with *flag-CaMKIIα, Pin1 WT, W34A*, and *R68/69A* plasmids were analyzed using a CaMKII kinase assay (Figure 3A). The co-expression of the Pin1 WT markedly reduced CaMKII total activity. The co-expression of the Pin1 mutant at the WW domain (Pin1 W34A) did not significantly reduce CaMKII activity. Pin1 mutant activity at the PPIase domain (Pin1 R68/69A) decreased even though suppression was weaker than in the WT. These results suggest that Pin1 suppresses CaMKII total activity, and that both the WW domain and the PPIase domain are required for this effect. Although the expression level of CaMKII was also reduced by Pin1 in the N2a cells (Figures 3B,C), the effect was not as pronounced as the effect on total activity. These results indicate that Pin1 suppresses CaMKII activity in N2a cells.

### Pin1 Reduced the Phosphorylation of Tau by CaMKII

**FIGURE 4:**
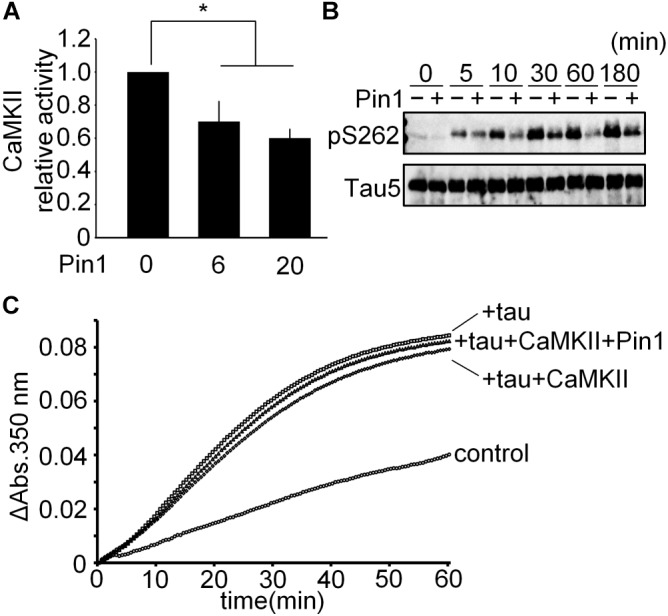
CaMKII-specific activity was suppressed by Pin1. **(A)** 5 nM CaMKII was incubated with 0, 6, and 20 nM Pin1 for 1 h, and an *in vitro* CaMKII kinase assay was performed using ATP-γ-32P. The data represent the means ± SDs of four independent experiments. ^∗^*p* < 0.05 using Dunnett’s test. **(B)** 10 μM recombinant tau was incubated with 10 nM CaMKII pre-incubated with or without 40 nM Pin1. The phosphorylation of tau was analyzed by western blotting using anti-tau pSer262 antibody, and Tau5 antibody was used for total tau. **(C)** 10 μM recombinant tau was incubated with 10 nM CaMKII with or without 40 nM Pin1 for 3 h. Non-phosphorylated tau was similarly incubated except for without CaMKII and Pin1. The sample (final 1 μM phosphorylated tau) was incubated with 15 μM tubulin at 37°C, and the absorbance at 350 nm was monitored for 60 min. Control indicates tubulin only.

To determine whether Pin1 suppresses CaMKII-specific activity, we performed a CaMKII kinase assay *in vitro* using baculovirus-derived recombinant CaMKIIα. CaMKII activity was reduced significantly by Pin1 (Figure 4A). We speculated whether Pin1 regulates the phosphorylation of tau by CaMKII. Recombinant tau was incubated with CaMKII with or without Pin1, and each phosphorylated tau was examined by western blotting using anti-tau pS262 antibody. As expected, Pin1 reduced the phosphorylation level of Ser262 of tau during at least 180 min (Figure 4B). It has been reported that phosphorylation of Ser262 reduces the interaction between tau and microtubules (Biernat et al., 1993). However, the microtubule polymerization activities of the three different kinds of tau prepared by the incubation with tau only, tau plus CaMKII, and tau plus CaMKII and Pin1, for 180 min were not different (Figure 4C). These results suggest that phosphorylation at Ser 262 is insufficient for tau to reduce microtubule polymerization as previously reported (Singh et al., 1996).

### Pin1 Rescued the Microtubule Polymerization Activity of CaMKII-Phosphorylated Tau

**FIGURE 5:**
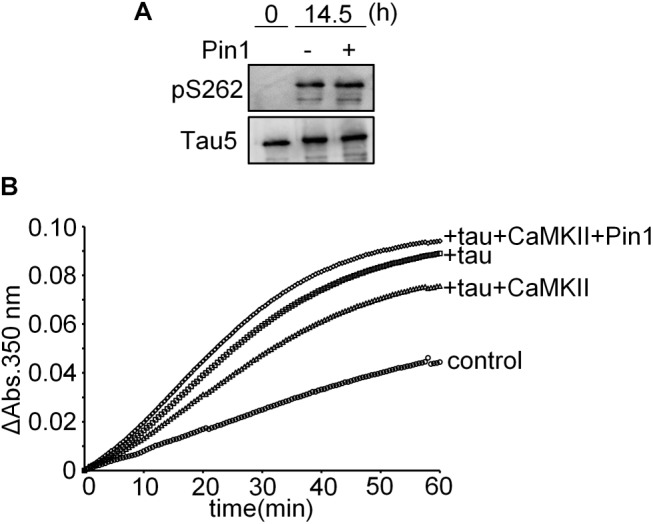
Pin1 reduced the effect of CaMKII on tau microtubule polymerization activity. **(A)** 10 μM recombinant tau was incubated with 50 nM CaMKII with or without 100 nM Pin1 for 14.5 h. Phosphorylated Ser262 tau and total tau were analyzed by western blotting using anti-tau pSer262 antibody and Tau5 antibody. **(B)** 10 μM recombinant tau was incubated with 50 nM CaMKII with or without 100 nM Pin1 for 14.5 h. Non-phosphorylated tau was similarly incubated except for without CaMKII and Pin1. The sample (final 1 μM phosphorylated tau) was incubated with 15 μM tubulin at 37°C, and the absorbance at 350 nm was monitored for 60 min. Three independent experiments were performed. Control indicates tubulin only.

We performed an *in vitro* microtubule polymerization assay using tau phosphorylated by CaMKII in the presence and absence of Pin1 for 14.5 h. Although presence of Pin1 did not change the phosphorylation levels of Ser262 of these samples (Figure 5A), the sample containing CaMKII-phosphorylated tau in the presence of Pin1 exhibited higher microtubule polymerization activity than that in the absence of Pin1 (Figure 5B). These results suggest that Pin1 enhances the activity of tau phosphorylated by CaMKII.

## Discussion

We have shown that Pin1 binds to CaMKII in the mouse brain (Tatara et al., 2010), but Ser/Thr-Pro site that phosphorylated by CaMKII has not been identified. CaMKII has four Ser/Thr-Pro sites, while Ser234 is the only phosphorylation site (Trinidad et al., 2008). Therefore, we speculate that Pin1 binds to phosphorylated Ser234-Pro in CaMKII. It is known that Pin1 binds to CaMKII, but it is not known the function of Pin1 for CaMKII. Since Pin1 decreases the activity of CaMKII, we speculated that Pin1 might decrease the amount of CaMKII or decrease its relative activity. By comparing the amount and activity of CaMKII in the brains of Pin1^-/-^ and WT mice, we found that the relative activity of CaMKII is suppressed by Pin1.

The dysregulation of signal transduction in the brain causes various neuronal functional disorders related to movement, language, and memory. Tauopathies and Alzheimer’s disease are caused by aberrant tau (Lee et al., 2001). Phosphorylation is closely related to aberration of tau and CaMKII also phosphorylates some functional sites of tau including phosphorylates Ser262 in the repeat domain of tau (Yoshimura et al., 2003), which reduces the affinity of tau to a microtubule (Biernat et al., 1993). These findings suggest that the activity of CaMKII profoundly affects the function of tau, although it has not been elucidated yet which phosphorylation site is the most important for tau activity.

Ser262 of tau was highly phosphorylated within 30 min. However, this phosphorylation did not decrease the microtubule polymerization activity of tau. On the other hand, the tau prepared by incubating with CaMKII for about 15 h reduced the activity of tau. These results show that phosphorylation of Ser262 is insufficient to decrease the affinity to microtubules as previously reported (Singh et al., 1996). We speculate that there are the other sites that are phosphorylated more slowly than Ser262 by CaMKII *in vitro*. It has been reported that Pin1 promotes the dephosphorylation of tau (Landrieu et al., 2011) and stabilizes microtubules via phosphorylated tau (Lu et al., 1999; Shimizu et al., 2017). Pin1^-/-^ mice had aberrant tau and degenerated axons in brains (Liou et al., 2003). However, the molecular mechanism behind the effect of Pin1 on microtubule polymerization is still controversial (Kutter et al., 2016). In our experiments, phosphorylation of tau was performed by CaMKII that is different from the kinase they used. Although we do not exclude the direct effect of Pin1 on the specific phosphorylated Ser/Thr-Pro site, we suspect that the total phosphorylation level of tau might induce the change of tau.

Calcium/calmodulin-dependent protein kinase II plays an important role in memory because it phosphorylates various substrates including the neurotransmission-related protein Synapsin I (Benfenati et al., 1992), AMPAR (Hayashi et al., 2000), and NMDAR (Sanhueza et al., 2011), as well as tau. CaMKII^-/-^ mice exhibit specific learning impairments (Silva et al., 1992), and aberrant CaMKII activity is associated with cognitive dysfunction in ADHD model rats (Yabuki et al., 2014). It has also been reported that Pin1^-/-^ mice have significantly enhanced late long-term potentiation (LTP) compared to WT mice (Westmark et al., 2010). Therefore, we speculate that Pin1 might play an important role in memory via Ca^2+^ signal pathways as well as neurological diseases like tauopathy and Alzheimer’s disease. We speculate that Pin1 might control many brain diseases by regulating CaMKII activity properly. In this report, we showed a novel mechanism of action of Pin1 to prevent neurodegeneration.

## Author Contributions

TU conceived the study and administered the overall study. TU and CU provided resources. TS, KK, and YS performed the experiments. TS and TU analyzed the data and wrote the manuscript.

## Conflict of Interest Statement

The authors declare that the research was conducted in the absence of any commercial or financial relationships that could be construed as a potential conflict of interest.

## Abbreviations

ADHD: attention deficit hyperactivity disorder
CaMKII: calcium/calmodulin-dependent protein kinase II
N2a: Neuro2a
*Pin1*^-/-^ mice: mice lacking *Pin1* gene
WT mice: wild type mice
